# Charitable Trusts and Charity Commissioners

**Published:** 1890-05-24

**Authors:** 


### The Hospital

A Weekly Journal of

Science, Medicine, Nursing, and Philanthropy.

*For Hospitals, Asylums, Medical Practitioners, Students, Nurses, Families, Parishes, Congregations, and the Charitable Public.*

Vol. VIII.—No. 191.] [May 24, 1890.

### CHARITABLE TRUSTS AND CHARITY COMMISSIONERS.

If a mountain claims the right to be tall it must admit the title of the adventurous to scale its loftiest peaks. Similarly when a man or a body of men place themselves or are placed on a conspicuous eminence before their fellow men they must also allow the right of their fellow men to criticise, to challenge, and even, if need be, to depose them from their eminence. The Charity Commissioners, so far as the vast mass of their fellow countrymen are concerned, live in the clouds; and charitable trusts, to the multitude, are ordinarily as remote from actual experience as the mysterious and unexplored depths of the southern seas. The British Parliament, in spite of its awkward feebleness, has sometimes the power of bringing distant things near, and of making those that are by nature hazy and uncertain, actual and present realities. A few days ago it summoned both Charity Commissioners and Charitable Trusts from the vasty deep, and made them appear for a brief season solid and palpable entities on the floor of the House.

In these days, when everybody is called upon to help to decide everything, it is desirable that everybody should know at least a little about everything. No one with a conscience will grudge a few moments to the consideration of Charitable Trusts and their relation to the Charity Commissioners. Parliament has passed the second reading of a Bill conferring additional powers upon the Commissioners. The debate upon the Bill covered the ground thoroughly, and the division proved that responsible men on both sides of the House were satisfied with the general principle of the measure. That of itself ought to convince the public that a reasonable thing is about to be done. However little mere party legislation may sometimes be worth, it is certain that when the best men of both parties are practically agreed, the course they decide upon can be justified both to the intellect and the conscience of the public.

Charitable Trusts constitute a property held by the public for the use of the poor and for other purposes. According to Mr. Jesse Collings there are in this country about 36,000 such Trusts; and they deal with the enormous annual income of over two and a quarter millions sterling. Now the very magnitude of this grand total cannot but be a striking object even to the least reflective. Those 36,000 charities may be likened to 36,000 orchards, each bearing a certain quantity of fruit annually, which must be annually dealt with, and the use of which may be highly beneficial, moderately beneficial, or partly beneficial and partly injurious to all persons who have anything to do with it. The question that naturally strikes any man who takes the least interest in the affairs of his country is—how may we best use those noble resources handed down to us by a generous past? Of course a very obvious reply is that the founders have in every case provided for the administration of their own bounty. Most true! But the wills of dead men can no more secure unqualified obedience than the laws of living kings; and it has therefore come about in many instances that monies left for charitable purposes have been to a large extent wasted, and sometimes even spent on objects which the benevolent founder, had he known, would have hated and detested. Here is a case in point instanced by Mr. Shaw-Lefevre in the course of the recent debate:—"There was," said Mr. Shaw-Lefevre, "the case of Brown's Hospital at Stamford, which had an income of £1,200 a year. Out of this only twelve alms-people were maintained; but there was a warder with a salary of £375 a year, and another official with a salary of £200 a year. Through the agricultural depression the income of the institution which was derived from rents had fallen off, and the trustees were advised to get rid of one of their expensive officials, but this they declined to do." That was one of the cases brought forward to show that though the machinery provided by the will of the founder was in full operation, yet the work done by it was not what the founder had ever for a single moment intended that machinery to do. He had left money to be placed in the hands of honorary trustees for the support of poor people in alms-houses. Instead of the trustees devoting, as they should have done, the whole of the money to the support of the poor, they spent, and probably are still spending, almost exactly half of it in the salaries of two highly paid officials. What duties those officials can perform which could not be done by an ordinary banker's clerk in his leisure time, and for which £50 a year would be ample remuneration, it is impossible to say. The Stamford case is an example of what may take place, and of what very often does take place, when charitable trusts are placed under the sole control of local trustees.

The Bill that passed its second reading a week or two ago is intended to remedy all this. Its object is to place practically all Charitable Trusts under the supervision of the Charity Commissioners. There is much to be said for such a proceeding, and something against it. But an impartial reader of the whole debate will almost certainly conclude that the step proposed to be taken is right and good. The case for the Charity Commissioners may be very briefly stated. Hitherto their control has been limited to trusts of smaller value than £50 a-year. But if it is desirable to control small trusts, say the supporters of the measure, is it not equally desirable that large ones should be placed under supervision? To that we should answer, as the House of Commons has answered, that in many cases it is. Some people will argue, as certain Members in the House argued, that local trustees will manage trust funds better than a central body. There is, however, no intention of choosing between local trustees and the Charity Commissioners. The latter are appointed to supervise the former, not to replace them: and the need for such supervision is, in many cases, shamefully evident.

Do even the proposers of the new measure desire to extinguish all local trustees by a wave of the Parliamentary hand, and to give to the Charity Commissioners unlimited control over all the charitable funds of the country? By no means! The Bill, which extends the range of the Commissioners' action without defining a limit, will, no doubt, become law; but checks and limitations will be imposed in Committee which will secure to local trustees their just rights. We desire to see the supervisory powers of the Commissioners extended, because, theoretically at any rate, they are the proper defenders of the interests of the poor, and of the intentions of the dead. Experience shows plainly enough that so far as local trustees are concerned "a living dog is better than a dead lion"; or, in other words, a living and greedy official has more influence with trustees than the written provisions of the dead testator. But the Charity Commissioners, being few in number, and carrying on their work at a central office, are, or ought to be, entirely independent of all kinds of ingenious local and official influence. They are like a central government or a court of judges. They have no particular interests to serve or personal ends to gain. Their sole duties are first to ascertain the will of the founder, and then to see that it is carried out as exactly as may be possible. Their interests are parallel with their duties, and there is, consequently, no temptation for them to go wrong.

As the measure before Parliament stands at present, there is practically no limit placed upon the range of the Commissioners' activities. That, as we have indicated, is a decided mistake. Whilst we agree that a financial limit is undesirable, we cannot admit either the necessity or the desirability of placing every kind of charitable organisation under the supervision of the Commissioners. There must be a principle of separation between charities. Such a principle is furnished by what may be called "living charities" as contrasted with charities whose resources have been furnished by the dead. As an example of what we mean by living charities may be mentioned voluntary hospitals, or hospitals which depend mainly or entirely for their support upon living persons. It is plain that such charities do not require the supervision of the Charity Commissioners; for the persons who furnish the charitable resources are still living and active, and they surely know best how they wish their charitable gifts to be administered. For the Commissioners, or any other body of men, to interfere in cases like these would clearly be an impertinence and an offence. Moreover, it would often prove injurious, and sometimes even fatal, to the very existence of the charities under consideration. Britons are very sensitive about interference with what they consider their private affairs; and it is certain that many of them would promptly cease to give any money at all to charities if they were not allowed the fullest possible liberty in the spending of it. In a word, then, whilst we welcome the extension of the supervisory control of the Charity Commissioners over Charitable Trusts properly so called, that is "dead trusts," we consider it imperative that voluntary charities, carried on and supported entirely or mainly by living persons must be absolutely left alone to the freest exercise of their own judgment and discretion.

